# Who learns more: the impact of dual-player and single-player modes in a serious game on dental students’ factual knowledge

**DOI:** 10.1186/s12909-024-05884-3

**Published:** 2024-08-21

**Authors:** Felix Krause, Ben Horn, Andreas Braun, Sebastian Fedrowitz, Laura Bell, Martin Lemos

**Affiliations:** 1 Department of Operative Dentistry, Periodontology, and Preventive Dentistry, RWTH Aachen University Hospital, Aachen, Germany; 2 Audiovisual Media Center, Medical Faculty, RWTH Aachen University, Aachen, Germany

**Keywords:** Dental education, Serious game, Factual knowledge training, Competition

## Abstract

**Background:**

The use of serious games in medical education provides a bridge between rapidly developing technology and traditional health-care teaching. Building on a promising web-based serious game for reviewing and acquiring factual knowledge in dental education, the present study investigated the benefits of a dual-player mode and various game options for enhancing knowledge gain and study motivation.

**Methods:**

Before the intervention, students’ dental knowledge and game experience were assessed using a pre-knowledge test and questionnaire-based self-assessment. Students in the clinical study phase (*n* = 57) were stratified based on prior knowledge and gender and then randomly assigned to two groups, with two player modes: single player (SP) and dual player (DP). In the SP group, each participant played alone, whereas in the DP group, the participants played against a previously determined peer. For a period of 4 weeks, the students were able to playfully acquire knowledge from the field of operative dentistry using METIS, a serious game application with three different game options (Marathon, Sprint, and Time). After the intervention phase, both groups completed a post-knowledge test. The usability of the serious game was evaluated with a self-assessment questionnaire.

**Results:**

The competitive game mode (DP mode; M = 8.69, SD = 0.45) resulted in an increase in the factual knowledge test that was a mean of 2.97 points higher than the SP mode (M = 5.72, SD = 0.43; *p* < 0.001). The DP group also found the game significantly more helpful for learning (*p* = 0.04) and engaged more with the teaching content because of the app (*p* = 0.04). Overall, the usability of METIS was rated as excellent, and students successfully improved their knowledge of dentistry after game play with both game modes (SP, DP, *p* < 0.001), with the game option “Marathon,” which involves playing the largest number of questions, being the most preferred.

**Conclusions:**

These results suggest that serious games such as METIS are a suitable educational medium for increasing students’ knowledge and interest in the field, and that competition with peers provides even greater motivation to engage with the learning content.

## Introduction

As (medical) education faces the dual challenge of creating attractive learning experiences and adapting to rapid technological advances, the gamification of educational content offers a promising bridge between traditional teaching methods and modern innovation. Through a symbiosis between entertainment and education, serious games — i.e., games designed for a purpose besides entertainment — seek to make learning as enjoyable an experience as it is for children [1]. Central to the creation of this type of attractive learning experience in serious games is the principle of motivation. According to the self-determination theory [2–4], a distinction can be made at the most basic level between intrinsic and extrinsic motivation. While the design and playful, pleasurable features of a serious game can serve as an intrinsic motivation to play, other types of game content such as feedback and rewards can serve as additional extrinsic motivators [4].

In line with this, previous studies have shown that serious games lead to an increase in individuals’ motivation to engage more intensively with the learning content, thereby increasing the time devoted to learning [5–8]. Accordingly, previous studies have shown that students in the medical and nursing professions generally show a positive attitude toward serious games [9, 10]. Reviews of published studies on serious games in health-care education show that serious games most often result in an increase in knowledge acquisition and are perceived as offering an attractive learning approach in comparison with traditional methods [7, 11–17].

Although they are not regarded as mainstream material in medical and dental education, serious games also provide tools for enhanced interactivity in health-care education, including dental education. During the pandemic, serious games were able to overcome the limitations of asynchronous learning [15]. While serious games have traditionally involved only a single player, two-player and multiplayer serious games have become increasingly popular in recent years. Incorporating competitive elements as an extrinsic motivator in educational games, as described by Cagiltay et al. [18], is a central strategy for enhancing both learning and motivation. This is important in the development of serious games, as it suggests that including competitive elements can significantly enhance the learning experience. Another study has added depth to this by differentiating the impacts of real versus virtual competition (where the participant plays against a computer). They found that real competition results in a high “flow” experience — involving complete absorption in and concentration on the task — which leads to better learning performance [19, 20]. It is notable that, despite the growing interest in and promising potential of serious games in broader medical education, the field of dental (theoretical) [7, 8, 21–25] education only recently has gained more interest and is still relatively underexplored [7, 8, 21–25] (see also for a recent systematic review by Sipiyaruk et al. [26]). A recent study highlights the potential motivating factor of quiz games in dental education [27]. However, no direct correlation was established with the actual increase in knowledge, e.g. in examinations. Another recent study by Lemos et al. aimed to explore this link further, looking at knowledge tests before and after serious game play. The study suggests that a serious game in single-player mode appears to be just as effective as traditional paper-based methods to acquire factual knowledge during the pre-clinical study period. It was shown that using the serious game was met with strong acceptance by the students, who reported that they spent more time with the teaching material than they did using traditional methods [8].

Building on these promising results with serious games for reviewing and acquiring factual knowledge in dental education and the benefits of competition, the aim of the present randomized controlled trial was to investigate the effect of further gamification — i.e., the implementation of a competitive element, as well as various game options — in further improving dental students’ acquisition of factual knowledge. 

The working hypothesis was that implementing a dual-player (DP) mode, as well as various game options (Marathon, Sprint, Time), would lead to an increase in students’ knowledge gain and motivation in comparison with a single-player (SP) mode. The user-friendliness of the application, new game modes and options, and the students’ usage behavior while playing were also evaluated.

## Materials and methods

### Study design

**Fig. 1:**
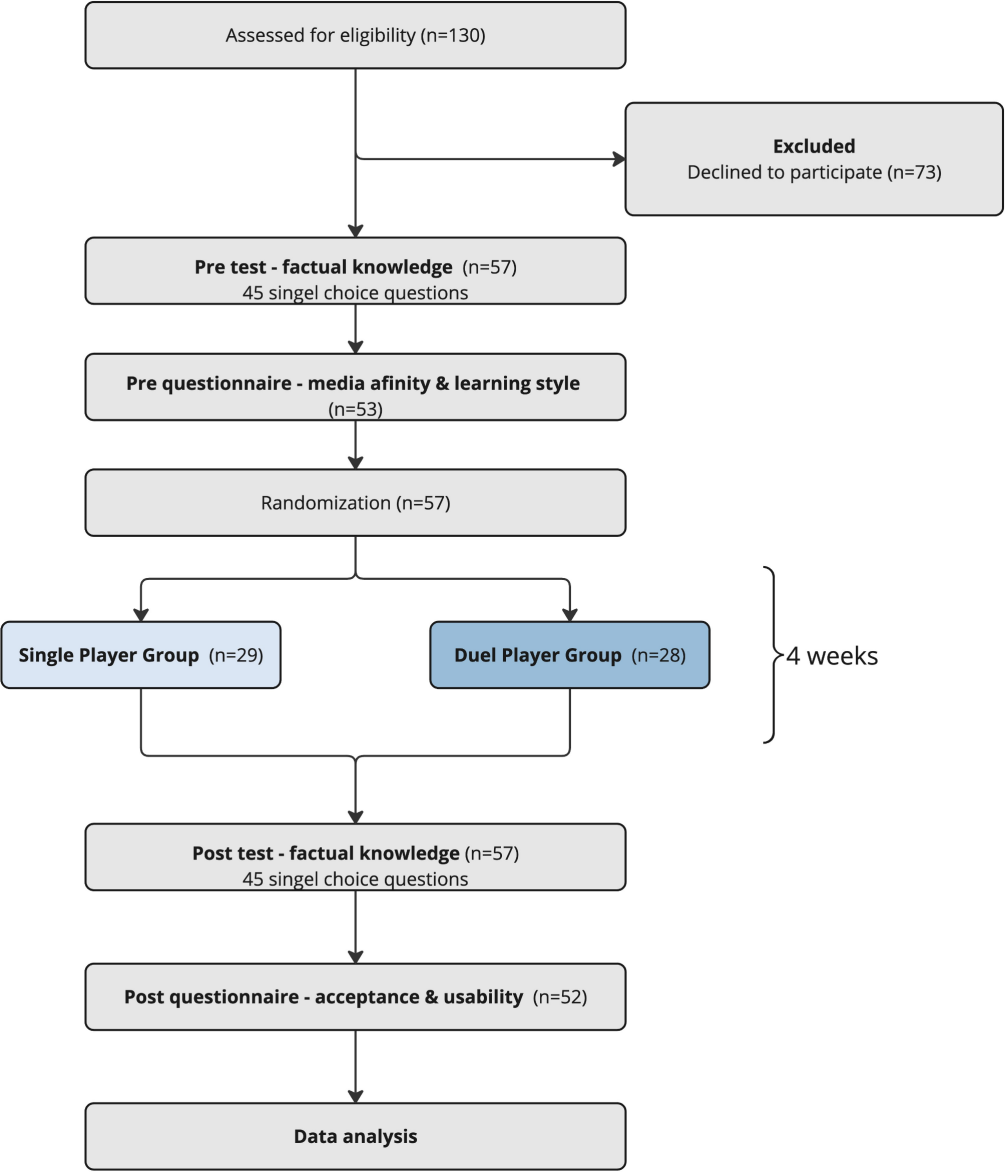
Study flow chart

The present study was performed as a randomized, single-blinded controlled trial with a pre-test (baseline) and post-test design. The study was anonymous, single-center, prospective, and comparative. Figure 1 provides a study flow diagram (Fig. 1).

### Participants

Dental students in their clinical study phase (the fourth and fifth years of study) in the summer semester of 2022 (start of April to middle of July 2022; *n* = 130) were asked to participate in the study on a voluntary basis. No exclusion criteria were specified. The students provided written informed consent to participate in the study. In the potential respondent group, a total of 57 students agreed to participate.

### Randomization procedure and group allocation

In accordance with the results of a formative pre-knowledge test (see the section on the outcome measure for factual knowledge, below), the 57 students were divided into two groups — a single-player (SP) group and a dual-player (DP) group, with half of the students in each group. A total of 29 students were allocated to the SP group and 28 to the DP group. The participants were allocated according to their gender and level of knowledge at the start of the study to ensure comparability and homogeneity of the test groups. In the DP group, fixed pairs were formed, taking the level of knowledge into account to ensure homogeneous pairs.

### Data protection and anonymization

Anonymous identifiers were created to link students’ responses across the study material. Before the start of the intervention phase, the students received anonymized, randomly generated identities (IDs) and passwords. An “identifier” program developed by the Audiovisual Media Center in the Faculty of Medicine of at RWTH Aachen University was used for this purpose. This allows the generation of identity–password combinations — e.g., from an e-mail address and the name of a childhood best friend.

### Serious game application

The web-based serious game METIS was programmed, designed, and developed by the Audiovisual Media Center at the RWTH Aachen University for the purpose of knowledge repetition and acquisition of factual knowledge [8]. The serious game can be used with many different devices — e.g., smartphones, tablets, or in the browser on desktop computers and laptops.

**Fig. 2:**
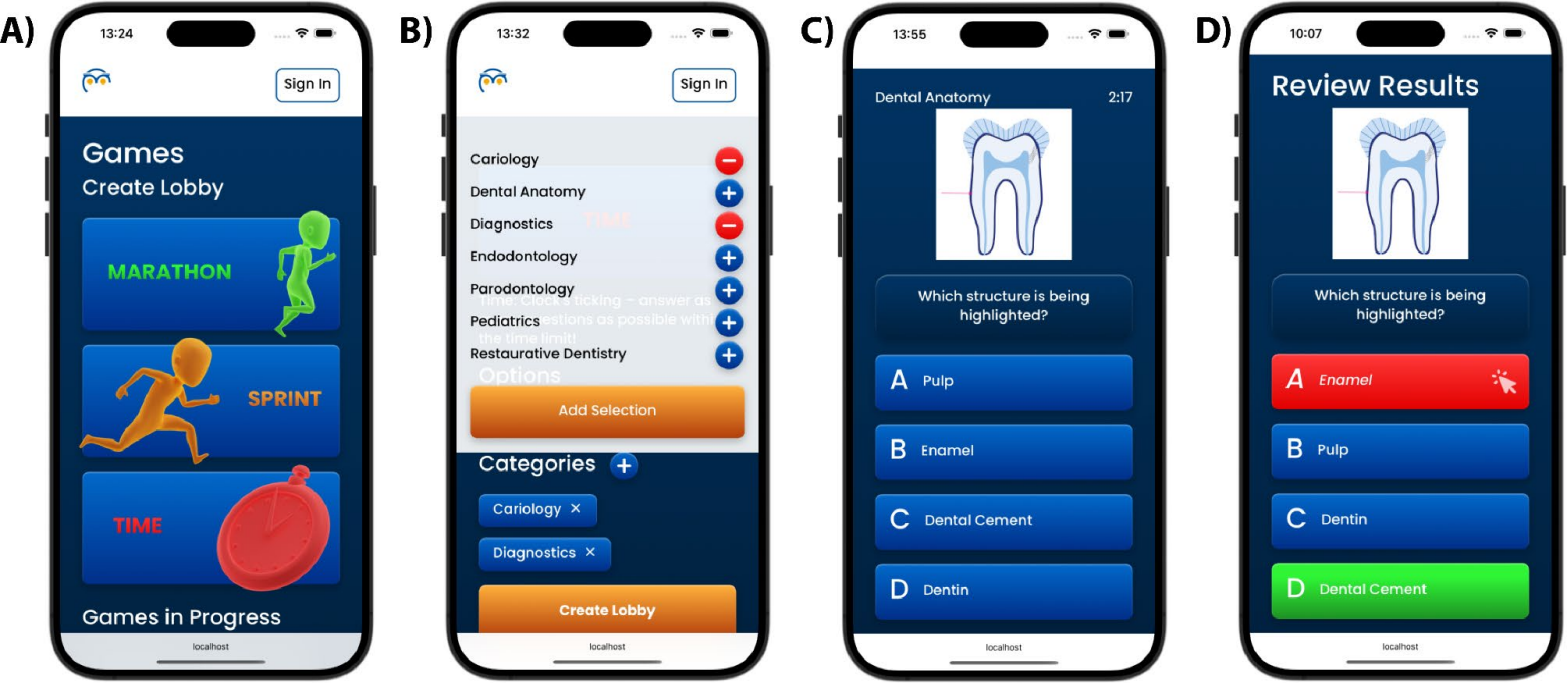
The serious game application METIS. **A** Screenshot of the home screen for the serious game. **B** The range of dental topics integrated into the game. **C **Screenshot of an example image question. **D** Screenshot showing an example of the review results

METIS is based on a question catalogue with 470 questions, which were created using a single-choice design by two dentists (BLH and FK), one of whom has extensive experience and additional qualifications in medical education (FK). The question catalog covers the subject areas of dental anatomy, cariology, restorative dentistry, endodontology, periodontology, dental diagnosis, and pediatric dentistry. The questions consist of a question stem (with or without illustration) and four different answer options (Fig. 2). Before the study was conducted, the question catalog was checked for plausibility and appropriate difficulty by two dentists from the Department of Operative Dentistry, Periodontology, and Preventive Dentistry who were experienced in teaching but not involved in the study.

In METIS, students were able to choose between the following game options:

- “Marathon”: all questions in the preselected topic categories can be played in one go.
- “Sprint”: students select the number of randomized questions in the preselected topic categories that they would like to play under time pressure in order to set a best time.
- “Time”: students answer as many questions in the preselected topic categories as possible within a defined and preset time limit.

Depending on which game option was selected, the students automatically received feedback on the questions played after a game session, or after each question. For more complex questions (e.g. with transfer knowledge, clinical reference or very specific factual knowledge), explanations of the correct answers were presented to improve the learning effect. No explanations were presented for basic factual knowledge questions (e.g., dental anatomy).

A game session always consisted of consecutive questions. On the results page, the students received feedback about how many of the questions in the game session were answered correctly, and they were able to revisit individual questions as they continued playing. In addition, their performance and progress in the game could be tracked, and the response time was recorded and displayed.

Importantly, the SP group only answered questions on their own without competing against a peer. The DP group played against a previously determined peer (see section on the randomization procedure and group allocation). Their game sessions got added to each other’s home screen for quick access. In addition, they could both see their respective cumulative scores on their home screen, facilitating the competition.

### Intervention process

For a period of four weeks, the students were advised to play the serious game as often as possible and with as many game sessions as they wanted. In the SP group, they played for themselves, while students in the DP group played against a predetermined peer student. While playing the game, user data such as player mode, game option, number of correctly and incorrectly answered questions, and points earned was collected.

### Outcome measure for factual knowledge

Pre-test and post-knowledge test scores were used as an outcome measure for improvements in factual dental knowledge. At the start of the study, the students completed a formative pre-knowledge test with a total of 45 questions from all areas of operative dentistry (dental anatomy, dental diagnosis, cariology, preventive and restorative dentistry, endodontology, periodontology, and pediatric dentistry). Each question in the test was based on a single-choice design with four answer options. One point was awarded for a correct answer per question, and no points were given for an incorrectly answered question.

After the four-week intervention phase, both groups participated in a formative post-knowledge test including 45 questions. To avoid repeating the questions asked (students could have looked up or discussed the solution between the knowledge tests), different pre-/post-knowledge test questions were chosen. In addition, the knowledge test questions were deliberately not identical to the quiz game’s question catalog but contained relevant learning objectives that were to be taught later in the serious game. Similarly, the subject areas in the pre- and post-knowledge test were kept similar so that students should be able to answer all questions with appropriate knowledge transfer. To ensure this, the difficulty level and focus of all questions were compared by two independent experts in the field. Both pre- and post-knowledge tests were conducted online via Dynexite (https://www.dev.dynexite.rwthaachen.de [in German; accessed January 10, 2024]) within a previously set fixed time frame (date and duration, 90 min). Dynexite is an examination software program developed by RWTH Aachen University that can be used to accompany the entire examination process — from implementing the questions, to compiling and conducting the examination, to publishing the results.

### Pre- and post-questionnaire

Students answered the pre- and post-questionnaires online using the open-source survey tool LimeSurvey (LimeSurvey GmbH, Hamburg, Germany; http://www.limesurvey.org). The pre-questionnaire concerned general questions on game usage, such as previous (serious) game knowledge, frequency of game play in leisure time, and game devices, as well as learning behavior and preferences — i.e., tendency to learn alone or in groups, openness to educational games and online quizzes. The post-questionnaire assessed the usability of the quiz app with the System Usability Scale (SUS) [28–30] as well as the short version of the User Experience Questionnaire (UEQ-S) [31]; preferences and acceptance of the different game options and modes; as well as game usage (where, which device, how often, how long the sessions were); and general feedback and suggestions on the serious game. Open-ended text questions were used to assess general feedback, and agreement with several questions about the app was rated using a 5-point Likert scale (1, strongly disagree; 5, strongly agree) or 7-point Likert scale (–3, fully agree with the negative term; 3, fully agree with the positive term). It should be noted that different Likert scales were used, based on the standardized questionnaires (SUS, UEQ-S); all remaining questions used a 5-point Likert scale. In addition, preferences for learning methods, game options, and modes were assessed using ranking questions.

### Data analysis

All statistical analyses were performed using the R statistical package (R Core Team, 2021). The significance level was set at α = 0.05. A linear mixed model (using the “lme4” package) [32] was used to ascertain the effects of the intervention (game play) on knowledge test results across the two modes (DP group vs. SP group). The dependent variable was the pre- and post-factual knowledge test results, with time (pre-, post-) serving as the within-subjects factor and player mode (SP, DP) as the between-subjects factor. Interaction terms for these factors were included in the model. Random intercepts for subjects were included to account for repeated measures on each participant. To control for possible confounding effects, gender (male, female) and semester (7th, 8th, 9th, 10th) were included as additional between-subjects factors. Post-hoc analyses examining interactions between time and group were conducted using Wilcoxon rank sum tests. Post-hoc analyses of the main effects were conducted using the estimated marginal means (“emmeans”) package [33]. The Tukey method was applied to correct for multiple testing. Wilcoxon rank sum tests were performed (using the base “stats” package) to explore potential differences between the game modes (SP, DP) or the game options (Marathon, Sprint, Time) in the questionnaires or the game itself, taking the skewed distribution of several items into account. The “rcompanion” package was used to calculate the effect size *r* [34]. The “ggplot2” package was used to visualize the data.

## Results

### Participants and their experience and openness to serious games

Fifty-seven dental students (female *n* = 35, male *n* = 17, NA *n* = 5) with a mean age of 25.21 years (SD 4.35) took part in the study. The students were in their seventh (30.8%), eighth (17.3%), ninth (25%) or tenth (26.9%) semesters. All students (*n* = 57) completed the pre- and post-knowledge tests and played the serious game. Fifty-three students completed the pre-questionnaire, and 52 students filled out the post-questionnaire.

While almost half of the students (47.37%) stated that they generally do not play games each day in their leisure time, around half of the students (50.9%) stated that, when playing games, they primarily use their smartphones. Interestingly, knowledge quizzes (30.2%) were played most, followed by puzzles (28.3%).

However, despite their interest in quizzes, the most frequent learning methods for all students were lecture slides and notes taken during lectures, recordings of lectures, and slides of lectures. The students also expressed a preference for studying alone, followed by studying in pairs and finally in study groups.

Although they showed a high level of agreement with using old exam questions for learning (mean = 4.17 out of 5; SD 1.46), the students were generally open to using online games to enhance and manifest their knowledge (mean = 3.94 out of 5; SD 1.35), with only 11.3% and 3.8% (completely) denying that they were open to using online games for study purposes.

### Improvement of factual dental knowledge

**Fig. 3:**
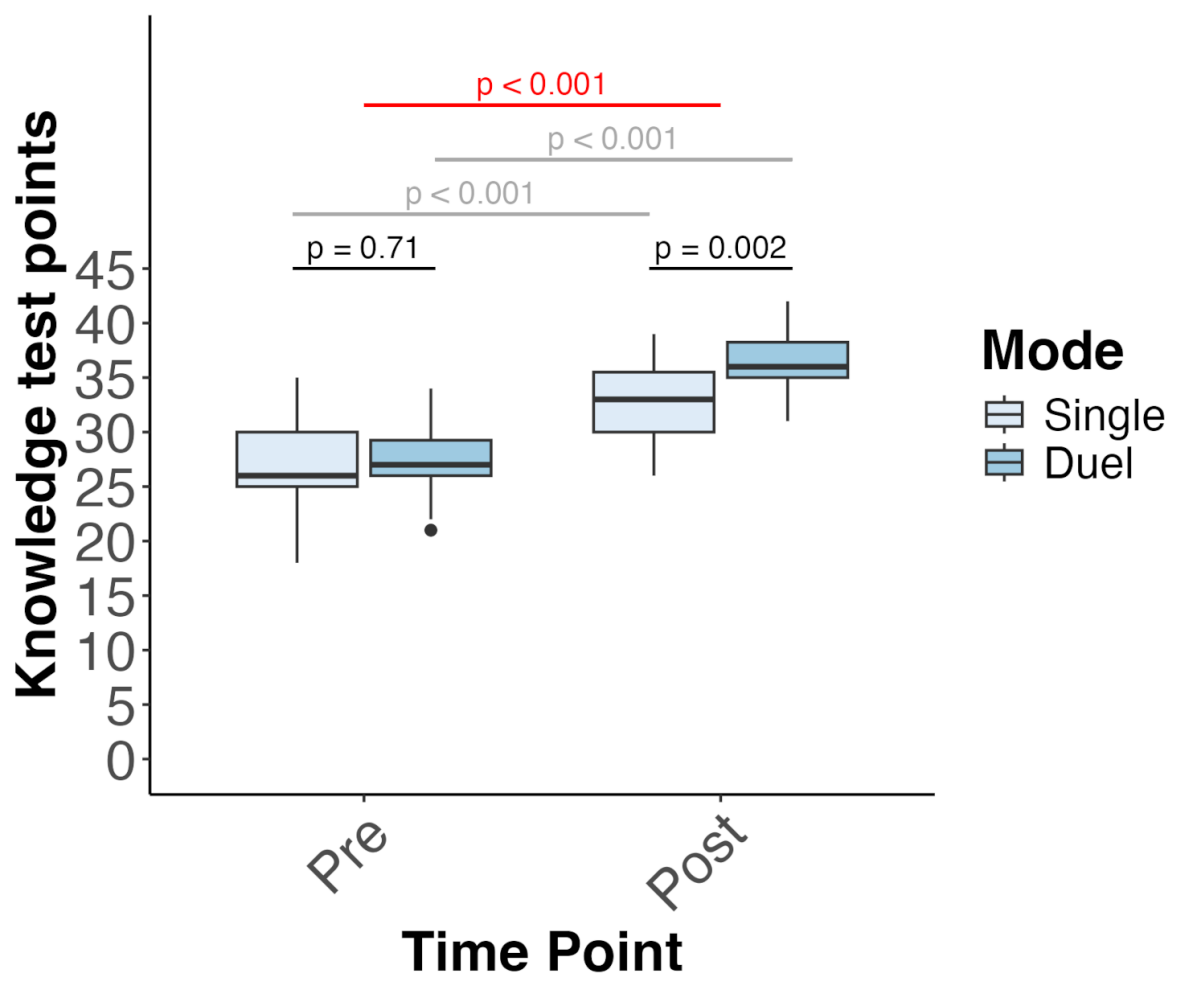
Significant improvement in factual dental knowledge, both in the single-player (SP) group and dual-player (DP) group, with a 2.97-point greater knowledge increase in the DP group than in the SP group. The box plots show median, first and third quartiles, and minimum and maximum values. The red line shows the significant interaction effect (mode, time point), gray lines show the significant major effects of time (pre-, post-), and black lines show the main effect of mode (single, dual)

**Fig. 4:**
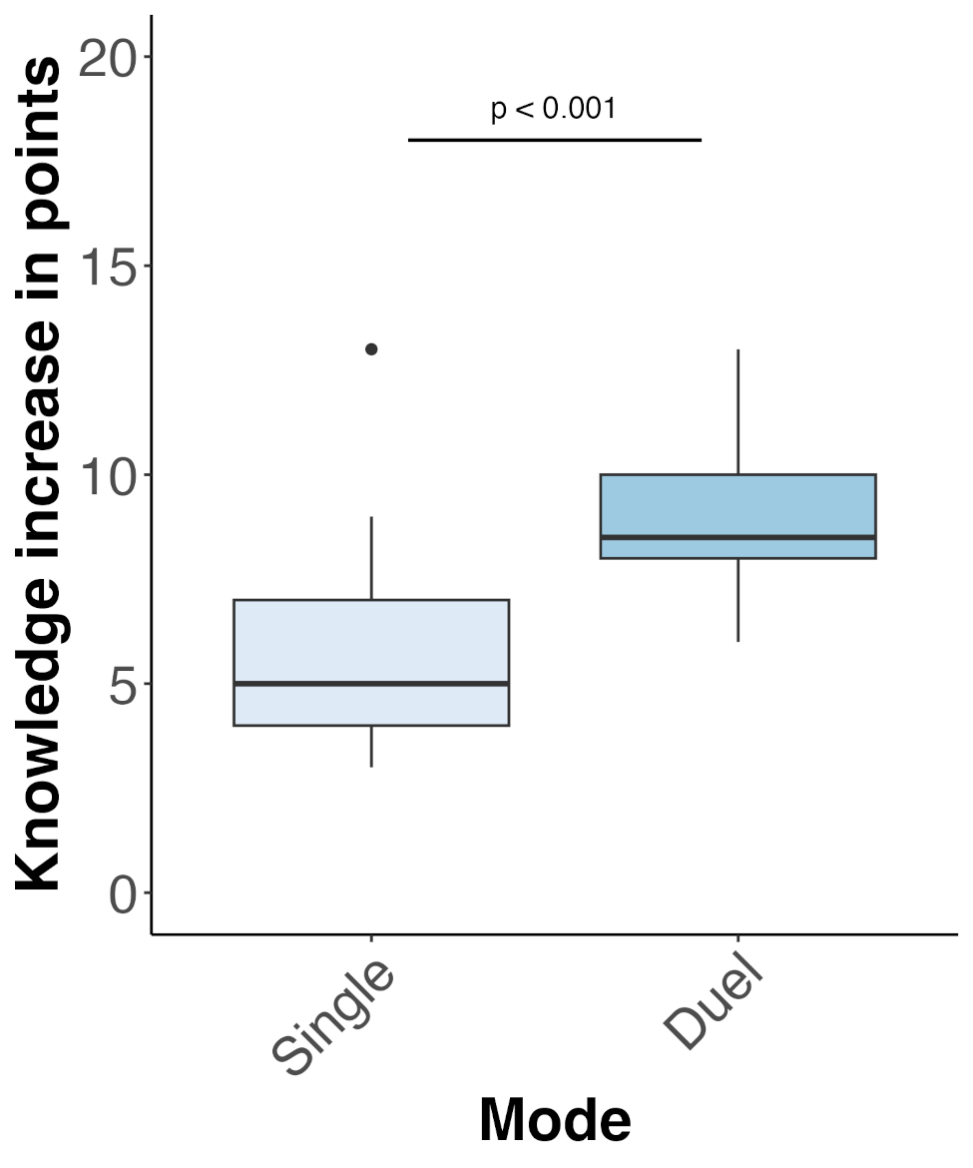
Greater improvement in dental knowledge in the DP mode in comparison with the SP mode

**Fig. 5:**
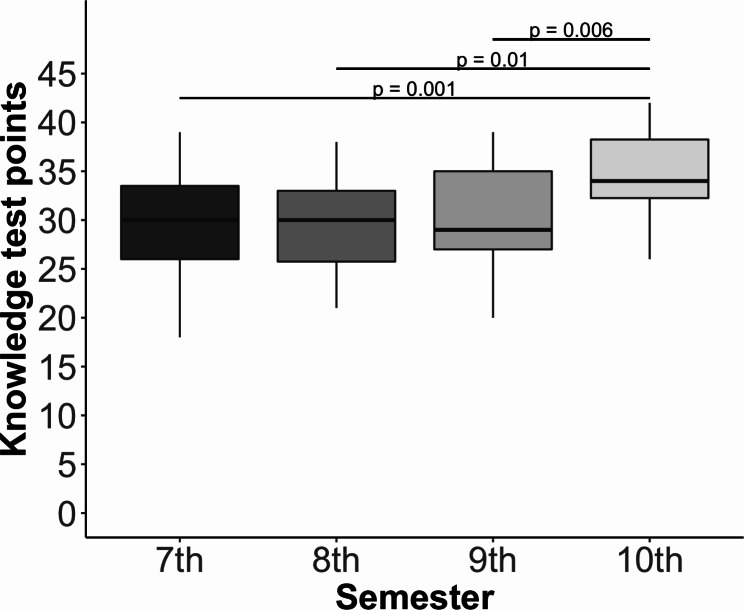
Differences in knowledge between the study semesters. The box plots show median, first and third quartiles, and minimum and maximum values

There was a significant main effect of time (pre-, post-knowledge test; *F*(1,49) = 668.03, *p* < 0.001, partial eta^2^ = 0.93), with both groups demonstrating an improvement in knowledge test results between the pre-intervention and post-intervention periods (Fig. 3). Importantly, a post-hoc power analysis conducted using the *boot* package [35, 36] indicated that the study had sufficient power to reliably detect the interaction effect (power = 0.99). In addition, a main effect of the player mode (DP, SP) was observed (*F*(1,45) = 5.83, *p* = 0.02, partial eta^2^ = 0.11), with the DP group outperforming the SP group, irrespective of the timing of the intervention (Fig. 3). Crucially, a significant interaction was observed between player mode (DP, SP) and time (pre-, post-knowledge tests; *F*(1,49) = 26.72, *p* < 0.001, partial eta^2^ = 0.35; Fig. 3). Post-hoc analyses revealed that although there were no differences between the two groups with regard to pre-intervention knowledge (Wilcoxon = 344, *p* = 0.71, *r* = 0.05), and both groups showed improved post-intervention knowledge (Wilcoxon = 488.5, *p =* 0.002, *r* = 0.44; Fig. 3), the size of the improvement was 2.97 points larger for the DP mode in comparison with the SP mode (*F*(1,45) = 25.59, *p* < 0.001; Fig. 4). In addition, there was a main effect of semester (*F*(3,45) = 6.62, *p* < 0.001, partial eta^2^ = 0.31). While students in all semesters improved their knowledge, students in their seventh, eighth, and ninth semesters knew significantly less than students in the tenth semester, irrespective of the intervention (Fig. 5). Finally, gender did not affect factual dental knowledge (*F*(1,45) = 0.45, *p* = 0.51, partial eta^2^ = 0.01).

### Self-perception of the acquisition of factual knowledge

Interestingly, in line with the knowledge tests, the students also subjectively experienced the serious game as being helpful for learning factual dental knowledge. Again, as with the knowledge tests, students in the DP group (mean = 5.42 out of 6, SD 0.97) found that the serious game was more helpful than students in the SP group (mean = 4.85 out of 6; SD 1.08, Wilcoxon = 416.0, *p* = 0.03, *r* = 0.28). In addition, students in the DP group also subjectively engaged more with learning content through the app (mean = 4.83 out of 6; SD 0.92) than the SP group (mean = 4.00 out of 6; SD 1.47; Wilcoxon = 418, *p* = 0.04, *r* = 0.28) and was trend wise more motivated by the app to study (mean = 5; SD 0.88; Wilcoxon = 407, *p* = 0.06, *r* = 0.25) than the SP group (mean = 4.27; SD 1.40). Although both rated the app as motivating to study (mean = 4.62 out of 6; SD 1.23).

### Usability of the serious game application

The general usability of the app was rated on the boundary between good and excellent, given the SUS score of 80.00 (*SD =* 14.42). The pragmatic and hedonic qualities, as well as the overall EUQ score, were rated as good (pragmatic mean = 1.66, hedonic mean = 1.34, overall mean = 1.50) and a German school grade of 2.1 (SD 0.71) was given (with 1 being the best grade and 6 the poorest). Interestingly, students in the DP group (mean = 1.88, SD 0.45) graded the game slightly better than the students in the SP group (mean = 2.31, SD 0.84, Wilcoxon = 218, *p* = 0.04, *r* = − 0.28). They also agreed slightly more that learning with the serious game was fun (mean = 5.21 out of 6; SD 0.59) than the SP group (mean = 4.50 out of 6; SD 1.27, Wilcoxon = 408, *p* = 0.049, *r* = 0.26) and found that the feedback from the serious game was more helpful (mean = 5.58 out of 6, SD 0.72) than the SP group did (mean = 4.85 out of 6; SD 1.26, Wilcoxon = 425, *p* = 0.02, *r* = 0.32). Nevertheless, all students strongly agreed that they had used the serious game to study (mean = 4.54 out of 6; SD = 1.33) and more than half of the students (55.92%) agreed that they did not experience the serious game play as learning. The general appreciation of the serious game and its motivational aspects were similarly reflected in the open-answer questions, e.g.:

- “The game provides a great time-saver for learning when the exam knowledge is specifically presented in the quiz.”
- “The quiz character means that this acquisition of dry knowledge is not perceived as learning.”

Comments on negative aspects were mainly concerned more with questions in the serious game itself or with minor technical issues that could be easily adjusted in a revised version of the serious game, in order to increase usability further, e.g.;

- “The loading time of the questions always took a little while, which was particularly annoying in time mode” // “In time mode, the app often hung up for a long time and the clock kept ticking” // “Images load slowly”.
- “The app was not a real app, but rather a website” // “web-based app”.
- “I really think the app has potential if the bugs that interrupt the flow of the game are fixed and new questions and quiz categories are constantly added. I can imagine using the app to increase and test my knowledge in the future!”

### User behavior

During the 4-week game-playing period, the students used the serious game primarily at home (followed by public transport, at university, or outside in their leisure time) and on their smartphone (62%). Almost half of the students (45.61%) stated that they used the serious game several times per week, and 10.53% even used the serious game once a day. While most game sessions lasted around 15–30 min (33.33%), game sessions ranged between less than 5 min and more than 1 h, since students were allowed to play the game in their own time and at their own pace.

The DP group earned slightly, but significantly, more points within the game (mean = 68.30, SD 9.67; 73% of the questions answered correctly) than the SP group (mean = 68.11, SD 9.81; 72.64% of the questions answered correctly) by answering questions correctly (*F*(1,52) = 5.84, *p* = 0.02, *f*^*2*^ = 0.003), despite answering fewer questions in total (*F*(1,52) = 3585.82, *p* < 0.001, *f*^*2*^ = 68.96).

Students preferred to use the Marathon game option (played in 84.4% of the game sessions), followed by Time (played in 10.6% of the game sessions) and least preferred to use the Sprint option (played in 5% of the game sessions). This was consistent with the ranking of the game modes in the post-questionnaire.

## Discussion

The main aim of this randomized and controlled study using a pre–post knowledge test design was to investigate the effect of competition (SP vs. DP mode) in a serious game on the acquisition of factual knowledge in the field of operative dentistry by undergraduate dental students. The effect of several game options (Marathon, Sprint, Time) was also explored.

### Motivational aspects and knowledge increase with serious games

Overall, the study indicated that students highly appreciated the serious game METIS across all game modes and game options, and experienced it as being helpful and motivating them to learn. This is consistent with the findings of other studies on serious games, demonstrating an increase in learning satisfaction and a positive attitude for students who use serious games as a learning medium [37–42]. The students were able to increase their knowledge across game modes and game options. This is generally in line with previous studies that have investigated the educational effect of serious games [7, 11–13, 15–17], as well as with findings from the previous version of METIS [8].

Students in their tenth semester showed higher knowledge scores in comparison with students in earlier semesters. This might be due to a higher level of initial knowledge and increased motivation to learn factual content, as tenth-semester students soon have to sit their final exams.

### Competition

Most importantly, this study suggests that the DP group had an advantage in comparison with their peers who played alone (the SP group): the extent of the knowledge increase after using the game was greater in the DP group than in the SP group. The study’s working hypothesis was therefore confirmed. Students in the DP group found that the serious game was more helpful than students in the SP group, and they also subjectively engaged more with learning content through METIS, which may have added to the larger extent of their knowledge increase. These results are consistent with findings in the existing literature, which suggest that competition in serious games enhances both learning and student motivation [18, 43–45]. Interestingly, previous research has shown that educational games also highly benefit from collaborative environments [46–48]. A study by Arayapisit et al. (2023) has for example shown that, next to competition, students benefited greatly from working together in a board game to learn to recognize the patterns of odontogenic infection, which led to students learning from their peers and ultimately to an even greater increase in knowledge. A similar cooperative condition for METIS, in which for example complex systems or concepts must be explained and discussed in small groups, could be investigated in the future to further explore this aspect in quiz games.

### Self-determination theory: autonomy (game modes), relatedness (competition), competence (feedback)

By adding a DP mode and various game options, METIS addresses the three intrinsic needs for autonomy, relatedness, and competence (a need for a challenge, to acquire new skills, knowledge, or abilities, or to receive positive feedback) (self-determination theory; [4].

In other words, firstly, the choice between different game options may offer students a sense of control (autonomy). METIS includes three game options (Marathon, Time, and Sprint) to allow the content to be learned in a playful way. “Marathon” was the most frequently played option here, followed by “Time” and “Sprint,” and this was mirrored by the students’ preferences. The “Marathon” option in METIS was probably most often played due to its design, which may have been perceived as less intensive and more engaging over longer periods. The marathon-style game allowed students a more individualized pace, enabling them to absorb information without the pressure of strict time limits or the high-speed nature of options like “Sprint” [49, 50]. The preference for the Marathon over Time and Sprint options may reflect a preference for a more immersive and less pressured learning environment, which can enhance motivation and retention of the material.

Secondly, the DP mode adds a social component (relatedness) that may encourage competitive behavior between students. It has previously been reported that interaction such as competition between players is a critical element for effective games. Competition is seen as a well-structured learning activity that attracts students’ attention [51] and is a useful method to motivate learning [18]. Thus, competition may give students an extrinsic motivation, and as a result students may put more effort and time into current tasks [52].

Finally, answering questions correctly, as indicated by the feedback provided, and defeating the opponent potentially provides a sense of achievement (competence). Feedback helps players to track their performance by providing information about success and failure [53]. Games ultimately should reward players for their game progress [54, 55]. In METIS, the players are shown immediately after answering the question whether they were right or wrong, with the correct answer option being highlighted in green. Explanations of the correct answers are also provided in more complex cases when required. In addition, the number of questions that were answered correctly is displayed at the end of each game option. Since the students found that METIS was helpful for learning and experienced significant learning improvement relative to their study progress (seventh to tenth semesters), it can be assumed that the challenge was in line with the students’ abilities.

### Limitations and outlook

The present study provides valuable insights into the effects of competition and various game options in serious games, but it is important to take several limitations into account that may affect the generalizability of the results. Firstly, while the sample size was sufficient to explore the effect of competition on knowledge, the sample size was too small to split it into further groups to explore differences between the three game options (Marathon, Sprint, Time). Future research with larger samples would further enhance the robustness of the findings and would allow insights into potential differences between the game options. It should also be noted that it was possible for students in the DP group to test a game session on their own. Future research should take this into account — e.g., by preventing the DP group from playing in the SP mode, in order to allow clean group comparisons. Similarly, future research might explore diverse groups of participants to enhance generalizability of the presented findings, as the presented voluntary sampling might introduce a response bias. Due to the curricular constraints, however, it was not possible to offer the students different interventions in this study. Despite these limitations, the current study to the best of our knowledge provides the first insight into competition in serious games in dental education and thus offers a basis for future studies. In addition, different game modes such as a multiplayer mode, question types, leader boards and difficulty levels could be incorporated. With these improvements, METIS could serve as a tool in the field of digital medical education, providing tailored and effective learning experiences for a wide range of health-care professionals. Expanding the accessibility of the game to a wider, international audience is a next possible milestone. At present, METIS is only available in German, and the game’s screenshots have been translated solely for publication purposes. While it is not feasible to create an international, English-language, or non-German version within the current study, it shows promise for future research.

## Conclusion

In conclusion, the present study highlights the potential of competition in serious games, such as a quiz game, for educational purposes and provides valuable initial insights into the use of serious games in dental education. Notably, the potential of METIS might extend beyond dental education, providing a foundation for effective learning across the health professions, with future versions enhancing its educational impact. Thus, future research should address the current limitations, to enhance the robustness of the findings and extend the scope of these results.

## Data Availability

No datasets were generated or analysed during the current study.
